# An Overview of the Role of Mechanical Stretching in the Progression of Lung Cancer

**DOI:** 10.3389/fcell.2021.781828

**Published:** 2021-12-24

**Authors:** Fengying Gong, Yuchao Yang, Liangtao Wen, Congrong Wang, Jingjun Li, Jingxing Dai

**Affiliations:** ^1^ Department of Traditional Chinese Medicine, Nanfang Hospital of Southern Medical University, Guangzhou, China; ^2^ Guangdong Provincial Key Laboratory of Medical Biomechanics and Guangdong Engineering Research Center for Translation of Medical 3D Printing Application and National Key Discipline of Human Anatomy, School of Basic Medical Sciences, Southern Medical University, Guangzhou, China; ^3^ Shiyue City Community Health Service Center, Shenzhen Integrated Traditional Chinese and Western Medicine Hospital, Shenzhen, China; ^4^ Department of Laboratory Medicine, Nanfang Hospital of Southern Medical University, Guangzhou, China

**Keywords:** mechanical stretching, mechanotransduction, lung cancer, cancer-associated fibroblasts (CAFs), cancer microenvironment

## Abstract

Cells and tissues in the human body are subjected to mechanical forces of varying degrees, such as tension or pressure. During tumorigenesis, physical factors, especially mechanical factors, are involved in tumor development. As lung tissue is influenced by movements associated with breathing, it is constantly subjected to cyclical stretching and retraction; therefore, lung cancer cells and lung cancer-associated fibroblasts (CAFs) are constantly exposed to mechanical load. Thus, to better explore the mechanisms involved in lung cancer progression, it is necessary to consider factors involved in cell mechanics, which may provide a more comprehensive analysis of tumorigenesis. The purpose of this review is: 1) to provide an overview of the anatomy and tissue characteristics of the lung and the presence of mechanical stimulation; 2) to summarize the role of mechanical stretching in the progression of lung cancer; and 3) to describe the relationship between mechanical stretching and the lung cancer microenvironment, especially CAFs.

## Introduction

**FIGURE 1:**
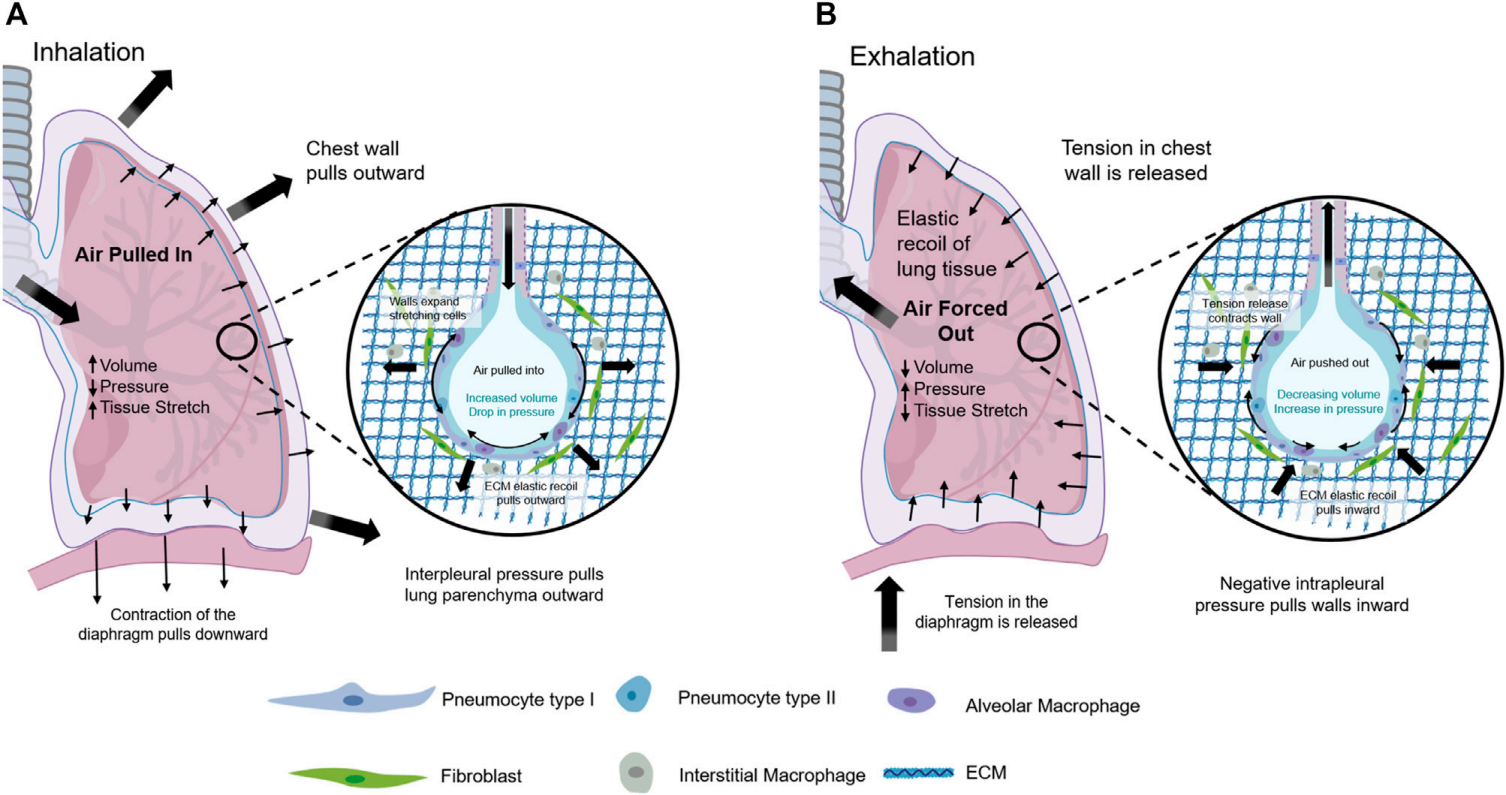
Mechanical Forces within the lung. **(A)** Contraction of the diaphragm and muscles in chest wall during inhalation led to negative interpleural pressure that enlarged lung tissue, stretched the alveoli, and increased lung volume driving air pulled in. **(B)** Relaxation of the diaphragm and muscles in chest wall during expiration permitted for elastic retreat that decreased lung volume and air compression that drove air forced out.

The main function of the lungs is to exchange oxygen and carbon dioxide with the outside world. The lung and thorax are important organs in the human respiratory system (Figure 1) (Novak et al., 2021). The respiratory muscles contract rhythmically, causing the thoracic volume to change periodically and then causing changes in pulmonary pressure, driving oxygen and carbon dioxide in and out of the lung to achieve pulmonary ventilation (Hsia et al., 2016; Doryab et al., 2021; Novak et al., 2021). Therefore, mechanical transduction plays a crucial role in lung health and disease. There are many types of mesenchymal cells in lung tissues. The fibroblast is one of the most important mesenchymal cells to maintain the normal physiological function of the lung. The heterogeneity of fibroblasts is responsible for their different phenotypes and activities (Kotaru et al., 2006).

Lung tissue is constantly influenced by breathing mechanics, and it is constantly exposed to a state of cyclical stretching and retraction. Therefore, lung cancer cells and lung cancer-associated fibroblasts (CAFs) are also influenced by mechanical load (Najrana et al., 2020; Roshanzadeh et al., 2020). Fibroblasts are the cells that make and sustain a structurally diverse array of ECM-rich connective tissues to support a wide-ranging of vital organ functions, like resistance to blunt and sharp damages in the skin or organ-wide stretching and flexible recoiling in the undamaged breathing lung. Three functions are generally assigned to fibroblasts: 1) secrete many of the same structural and signaling macromolecules that donate to the extracellular space; 2) adopt a transient and contractile myofibroblast phenotype in response to tissue injury; 3) play an important role in signaling extracellular cells for tissue-resident stem cells, or serve as mesenchymal stem cells, which can differentiate to specialized mesenchymal cells (Lemos and Duffield, 2018; Pittenger et al., 2019; Plikus et al., 2021). A main constituent of the tumor stroma is fibroblasts, and numerous studies have demonstrated a prominent functional role for these cells in cancer progression and metastasis (Kalluri and Zeisberg, 2006; Öhlund et al., 2014). Tumor related fibroblasts have been labeled CAFs, tumor associated fibroblasts, activated fibroblasts or activated myofibroblasts and could take in cancer-associated mesenchymal stem cells. To facilitate tissue repair, chemical and physical clues induce quiescent fibroblasts to myofibroblasts that secrete a lot of expressing contractile proteins in ECM such as α-SMA that coordinate biomechanical remodeling and contraction by traction (Plikus et al., 2017; Plikus et al., 2021).

Thus, to better explore the progression of lung cancer pathogenesis, it is necessary to combine cell mechanics factors to obtain a more comprehensive analysis. The purpose of this brief review is: 1) to provide an overview of the anatomy and tissue characteristics of the lung and the presence of mechanical stimulation; 2) to summarize the role of mechanical stretching in the progression of lung cancer; and 3) to describe the relationship between mechanical stretching and lung cancer microenvironment, especially CAFs.

## Physiological and Biomechanical Characteristics of the Lung

The lungs are in the chest cavity, on either side of the mediastinum, above the diaphragm. The lungs are composed of two functional areas: 1) the airways through which air enters and passes the pharynx, larynx, trachea, main bronchi, bronchioles, and terminal bronchioles; and 2) the respiratory zone (gas exchange zone), including the alveolar tubes, bronchioles, and alveolar sacs (Ahadian et al., 2018).

The main function of the lungs is to exchange gas with the outside world. Through the external respiration function of the lungs, oxygen (O_2_) is continuously provided to the body and carbon dioxide (CO_2_) is discharged to maintain the body’s blood and gas balance and internal environment in equilibrium. The alveoli, which provide a large surface area for gas exchange, are lined with a thin layer of epithelial cells that form a tight single membrane on the basement membrane (1.66 ± 0.128 μm) in normal lungs (Divertie et al., 1976) and are directly connected to the endothelial lining of the capillary network. The space between the epithelium and endothelial lining is called the lung interstitium and contains various cellular and extracellular matrix (ECM) components that provide structure and support for the lungs (Novak et al., 2021).

## Pathological Changes of Lung

In general, lung diseases fall into two categories: 1) restrictive diseases, which include a reduction in the ability of the lungs to expand and 2) obstructive diseases, which cause increased airway resistance and restricted airflow. Restrictive or obstructive diseases a classified based on clinical measures, including the ratio of forced expiratory volume to full forced lung capacity in one second (FEV1/FVC) and total lung capacity percentage (TLC%) (Kouranos et al., 2020). The restrictive disease is characterized by a decrease in the TLC% with no change or increase in the FEV1/FVC ratio because either both indicators decrease simultaneously or forced expiratory volume in one second (FEV1) increases with decreased lung compliance. Restrictive diseases, such as pulmonary fibrosis, interstitial lung disease, and sarcoidosis, often result from the accumulation of components of the ECM and scar tissue around the alveoli that affects the stiffness of the lung parenchyma and limits the lung’s ability to expand. Obstructive diseases, such as asthma, bronchopulmonary dysplasia, bronchiolitis obliteration, and chronic obstructive pulmonary disease, due to the degradation of lung connective tissue, lead to airway swelling, reduced matrix hardness and elasticity, and reduced lung retraction, and interfere with an individual’s ability to exhale adequately (Novak et al., 2021).

Pulmonary fibrosis (PF) is a chronic, restrictive lung disease in which excessive collagen deposition results from the accumulation of scar tissue in the lung caused by lung injury, inflammation, and/or long-term exposure to toxins or particles. Idiopathic pulmonary fibrosis (IPF) is a chronic progressive pulmonary fibrosis disease with unknown reasons. In recent years, more and more studies have shown that IPF is closely related to the occurrence of lung cancer. Also, IPF diagnosis and treatment guidelines in 2011 clearly indicate that IPF is prone to lung cancer, pulmonary embolism, pulmonary hypertension, and other lung diseases (Raghu et al., 2011; Raghu et al., 2015). According to the latest research statistics, the incidence of lung cancer in IPF population is 2.7–48% (Ballester et al., 2019), which is significantly higher than that of the general population (2–6.4%) (Bouros et al., 2002). Undissolved scar tissue in the lungs hardens the lung parenchyma and limits lung dilation. Matrix over deposition occurs in the distal airway structure, where fibroblast lesions are composed of excess collagen, fibrin, and other ECM components that are insoluble and deteriorate over time (Burgess et al., 2016). Heterogeneous lung stiffness results in increased stress and strain, affecting cellular mechanical conduction and disease progression. Tsukui et al. (2013) constructed an *in vivo* model of bleomycin-induced pulmonary fibrosis to study the gene expression profile of fibroblast populations. They found that osteopontin is highly overexpressed in lung fibroblasts and can serve as a marker of CAFs activation. It has been reported that biomarkers commonly expressed by CAFs include α -smooth muscle actin (α-SMA), fibroblast specific protein-1 (FSP-1), fibroblast activating protein α (FAP-α), platelet-derived growth factor receptor -β (PDGFR-β), neural/glial antigen (NG2) (Kalluri, 2016; Nurmik et al., 2019), and Gamma-glutamyltransferase 5 (GGT5) (Wei et al., 2020). Su S et al. reported CD10^+^/GPR77^+^ CAFs promoted cancer development and chemoresistance by sustaining cancer stemness (Su et al., 2018). Interestingly, osteopontin expression is significantly increased in senescent fibroblasts and is a key mediator of senescent stroma promoting tumor progression (Pazolli et al., 2009).

## Association Between Mechanical Stretching of the Lung and Lung Cancer

Cells respond to both chemical and mechanical signals in their microenvironment. Various mechanical stimulus signals (e.g., basal rigidity, hydrostatic pressure, compression, tension, and shear stress) are detected and transmitted to cells *via* mechanoreceptors. These receptors often encounter the ECM, where external signals are converted into physiological responses that affect cell proliferation, differentiation, and migration (Jang and Beningo, 2019).

### Studies on Cell Mechanics Involving Lung Tissue Cells

A major feature of the lung is its unique mechanical force. Each respiratory cycle imposes a periodic mechanical force, and this dynamic mechanical force exposes the lung to a challenging system of external reconstruction (Ahadian et al., 2018). The experiments by Tschumperlin and Margulies have shown that circulatory stretching stimulation of 5–12% is equivalent to 60–80% of the total lung volume and is associated with the physiological level of mechanical strain experienced by the alveolar epithelium and microvessels during low tidal volume mechanical ventilation, which is the protocol used in lung protection strategies (Tschumperlin and Margulies, 1998; Tschumperlin and Margulies, 1999). In contrast, circulatory stretching at 17–22% linear dilation is equivalent to 100% of total lung volume and is associated with pathophysiological conditions induced by mechanical ventilation of the organism volume and with inflammatory responses *in vivo* and with acute lung injury (Tschumperlin et al., 2000).

The lung is a dynamic organ with complex mechanical environment at microscale (Shikata et al., 2005). Thus, mechanotransduction plays a vital role in lung health and disease. The effects of mechanical force on proliferation, morphology, ECM composition, and alteration, gene regulation and the inflammatory response of lung cells and lung cancer cells have been recognized by most researchers (Novak et al., 2021). Nalayanda et al. (2009) observed that the growth rate of A549 cells decreased as the shear stress increased. However, Mahto et al. demonstrated that the effect of shear stress on alveolar cells was affected by cell species. The secretion of surface-active substances in human A549 cells showed no change below 8 dynes/cm^2^, but was impaired when it was above 8 dynes/cm^2^. The secretion of mouse MLE-12 cells increased with the growth of stimulation (Mahto et al., 2014). There are conflicting findings in the literature on the role of matrix rigidity on epithelial cell function. Eisenberg et al. (Jones, 2011) established that substrate rigidity affected cell morphology, microfilaments, and focal adhesion, but did not adjust the differentiation of EMT or cell types (from ATII into ATI) in mouse alveolar epithelial cells. In contrast, Marilyn M. Dysart et al. found that environmental particulate enhances stiffness induced alveolar epithelial cells mechanoactivation of TGF-β (Dysart et al., 2014). Markowski et al. (2012), reported that augmented matrix rigidity induced EMT, integrin binding, and TGF-β activation. The cell mechanics of lung cancer cells will discuss in the next section.

Many researchers have developed *in vitro* systems to detect the response of lung cells to mechanical forces. These models typically study how one type of mechanical force affects one cell type, and there is very little work combining multidimensional forces and multicellular models to accurately summarize the complex interactions that occur in the body. Typical devices *in vitro* mechanical stimulation involved: 1) cyclic uniaxial or biaxial, equi-biaxial strain for cell stretch stimulation (Felder et al., 2008; Rapalo et al., 2015) 2) static or cyclic pressure devices on cell stimulation (Huang et al., 2010); 3) microfluidic device for wall shear stress on cells (Mahto et al., 2014); 4) gradient stiffness hydrogel for cell culture (Liu et al., 2010); 5) cyclic strain device for capillary interface (Huh et al., 2010); 6) interstitial fluid flow custom device for cells seeded on the gel (Ng and Swartz, 2003). The effects of endothelial cells stimulated by shear stress have been widely studied. Shear stress is a known factor impacting endothelial cell morphology (Szulcek et al., 2016), cytoskeletal remodeling (Birukov et al., 2002), and Ca^2+^ levels in plasma (Yamamoto et al., 2018). Nitric oxide release is donated to maintain vasomotor action, anti-inflammatory mechanism, and cytoplasmic antioxidant ability (Tousoulis et al., 2012). Endothelial cells increased their ACE2 expression with pulsating shear stress stimulated, thereby enhancing the level of nitric oxide and decreasing proliferation and inflammation. ACE2 level was enlarged when endothelial cells were stimulated and stretched in the Flexcell device (Song et al., 2020).

### Studies on Cell Mechanics on Lung Cancer Cells

Like normal cells, cancer cells recognize the mechanical changes provided by the tumor microenvironment and convert them into signaling pathways through mechanical transduction pathways (Table 1) (Sporn and Albini, 2007; Xu et al., 2017). Wang et al. (2020) found that after cyclic stretching of the A549 lung cancer and IMR-90 fibroblast cells, cells were rearranged, the cytoskeleton was restructured, and an increased stretching time could prolong mitochondrial length. Weber et al. (Wang et al., 2020) found that various human lung epithelial cell lines could adapt to chronic cyclic strain stimulation. Hendricks et al. (2012) studied the effects of the simulated force of near-normal respiration (20% maximum strain and 15 cycles/min) on proliferation and morphology of NCI-H358 and A549 cell lines. They showed that mechanical stimulation reduced cell proliferation. Shukla et al. (2016) demonstrated that higher substrate rigidity induced slower and directional migration of lung cancer cells by decreasing the phosphorylated focal adhesion kinase and paxillin, not the biomarkers of EMT. Furthermore, Barenholz Cohen et al. (2020) demonstrated that tumor-derived extracellular vesicles from breast carcinoma cells caused the lungs of cancer-free mice broadly variable, and being more elastic than viscous. Matrix rigidity encourages microtubules glutamylation by increasing glutamine metabolism and strengthening microtubules stabilization, thus stimulating the migration of cancer cells (Torrino et al., 2021).

**TABLE 1:** Stimulation experiments of lung cancer cells by mechanical stretching.

Cell type	Mechanical stretching	Results	References
Lung epithelial cancer cells (A549)	Short-term stretching (15, 30, and 60 min) and long-term stretching (24 h), 10% cell surface area, 1 Hz, incubated at 37°C	Cell rearrangement, cytoskeleton reorganization, and increased stretching time can prolong mitochondrial length	Wang et al. (2020)
Fibroblast (IMR-90)			
AT II cell-like A549	16% surface elongation, 12 min^−1^	Several kinds of human lung epithelial cell lines can adapt to chronic cyclic strain	Wang et al. (2020)
serous glandular epithelial cell-like Calu-3			
NCI-H322			
NCI-H358			
Lung epithelial cancer cells (BEAS-2B)			
Lung epithelial cancer cells (A549)	20% maximum strain and 15 cycles/minute	Decrease cell proliferation	Hendricks et al. (2012)
NCI-H358			

### CAFs and Studies on Cell Mechanics

#### Fibroblasts and Mechanical Stimulation

The lung is a complex organ, and types of mesenchymal cells are found in its tissues. Fibroblasts are one of the most important mesenchymal cells that contribute to maintaining the lung’s normal physiological function. The heterogeneity of fibroblasts is the key reason for their different phenotypes and functions (Öhlund et al., 2014). Fibroblasts from different parts of the lung present significant differences and can be divided into different subgroups according to their heterogeneity (Chang et al., 2002; Kotaru et al., 2006). Kotaru et al. (2006) found that there are two kinds of fibroblasts at least: Airway fibroblasts (AFs) and distal lung fibroblasts (DLFs). AFs are larger, stellate-shaped with more cytoplasmic protrusions, and DLFs are spindle-shaped. AFs expressed more procollagen type I and eotaxin-1 than DLFs did at and after TGF-β treatment. In contrast, AFs had low proliferation rate than DLFs with serum treatment. Moreover, AFs expressed less α-SMA than DLFs in reference point. Pechkovsky et al. (2010) demonstrated results by comparing human proximal bronchi (B-FBR) and distal lung parenchyma (P-FBR). It indicated that P-FBR showed improved TGF-β/Smad signaling at the reference point, and the activated TGF-β significantly reduced basal a-SMA protein in P-FBR. Xie et al. (2018) segregated six subpopulations in adult pulmonary mesenchymal cells: myofibroblasts, Col13a1 matrix fibroblasts, Col14a1 matrix fibroblasts, lipofibroblasts, mesenchymal progenitors, and mesothelial cells.

Fibroblasts are cells that respond to mechanical stretching stimuli (Table 2). They maintain the structure and function of organ tissues by altering the expression of genes and proteins in their ECM in response to external physical (such as tensile force), chemical (such as chemical poisons), and biological (such as infectious toxins) factors (Amma et al., 2005; Sakamoto et al., 2010).

**TABLE 2:** Stimulation experiments of lung fibroblasts by mechanical stretching.

Cell type	Mechanical stretching	Results	References
Human embryonic lung fibroblast MRC-5	Flexcell FX-5000; mechanical tensile stimulation continuously for 48 h (0.1Hz; Sine waves, stretching amplitude of 5, 10, 15 and 20%)	Mechanical stimulation of 5% stretching increased cell proliferation. However, it had no significant effect on expression levels of TGF-β1 and collagen. Mechanical stimulation with 10% tensile force inhibited cell proliferation but increased expression levels of TGF-β1 and type I collagen. 15 and 20%, with significantly larger effects	Xie et al. (2020)
human lung fibroblasts	Strex ST-140; uniaxial tension (strain 10–30%); 30 cycle/min for 10 min	Mechanical stretching induces calcium influx and releases ATP independently of conventional stretch-sensitive ion channels, known as actin cytoskeleton	Murata et al. (2014)
human lung fibroblasts	Flexercell FX-4000; 0.2 HZ, maximum elongation 10%, 24 or 48 h of cyclic mechanical strain	The mRNA expressions of COL1A1, COL1A2, COL3A1, COL5A2 and Tenascin C were decreased. Cyclic mechanical loading on primary human lung fibroblasts for 48 h reduced the expression of fibrosis-related genes. Myofibroblast differentiation is reduced under these conditions. cyclic mechanical loading decreased the expression of endogenous TGF-β1	Blaauboer et al. (2011)
Mouse fetal lung fibroblasts: wild-type and EGFR knockout	Flexcell FX-4000; equibiaxial cyclic strain of 2.5% or 20% was applied at 40 cycles per min intervals for 48 h	Traumatic stretch (20% stretch) results in lactate dehydrogenase release at the same level in wild-type and knockout cells. EGFR does not alter the mechanical properties and damage resistance of fetal fibroblasts exposed to mechanical stretching	Giordani et al. (2013)
	A 2.5% stretching scheme was selected to simulate physiological stretching and 20% to simulate injury	20% stretching increased lysed caspase-3 and decreased proliferating nuclear antigen only in wild-type cells. 20% stretching increased macrophage inflammatory protein-2 and monocyte chemotactic protein-1 in wild-type cells. In knockout cells, miP-2 was reduced by 50% and McP-1 increased by only 60% compared to physiological stretching	
Mouse fetal lung fibroblasts	Flexcell FX-4000 Strain Unit; 20% cyclic stretch, 40 cycles/min for 48 h	After 24 h, LDH levels had increased by 50%. After 48 h mechanical stretching, fibroblast lysis increased	Hawwa et al. (2011a), Hawwa, et al. (2011b)

Pechkovsky et al. (2010) found that fibroblasts derived from the lung parenchyma cilia develop α-SMA, whose high expression characterizes a specific fibroblast phenotype. These cells are responsible for the composition of the ECM. Components, mainly cellular protein deposition, may be involved in the pathogenesis of idiopathic pulmonary fibrosis, and fiber proliferative disease and may play a key role in the occurrence and development of lung cancer. The cyclic load to which healthy lung cells are exposed during respiration prevents fibroblasts from differentiating into myofibroblasts (Blaauboer et al., 2011). suggesting that fibroblasts are static in their physiological state. Low levels of mechanical stretching may be important for interstitial remodeling during lung development. However, extensive stretching can damage the normal lung structure (Hawwa et al., 2011a; Sanchez-Esteban et al., 2002).

After the lung tissue is slightly injured, fibroblasts in the lung parenchyma differentiate into myofibroblasts (Tomasek et al., 2002) to promote wound healing. After tissue repair, apoptosis of myofibroblasts occurs once the remodeling balance of repaired tissue is restored. During fibrosis, however, myofibroblasts remain active after wound healing, producing an excess of strongly contracted ECM components. Xie et al. (2020) showed that (5–10%) mechanical stimulation could improve the proliferative activity of MRC-5 cells and may slightly stimulate the expression of transforming growth factor-β (TGF-β) 1 and type I collagen in human embryonic lung fibroblasts. Mechanical stimulation (15–20%) directly leads to cell damage, reduces cell proliferation activity, induces TGF-β1 expression, significantly increases collagen expression, and accelerates the process of pulmonary fibrosis.

After mechanical stimulation at different tensile ranges, the biomechanical properties of MRC-5 fibroblast cells decreased. And, the fibroblasts appeared to “soften”, indicating that the degree of deformation of lung fibroblasts was lower than the linear stress-strain relationship. This feature is not a reflection of a specific molecular mechanism but indicates higher-level structural changes, which suggests that the cytoskeleton may have been damaged (Xie et al., 2020). Other *in vitro* studies have shown that mechanical stretching with higher strain ranges (20–30%) activates the Ca^2+^ influx pathway independently of the actin cytoskeleton and the conventional stretch sensitive ion channel (such as members of the transient receptor potential (TRP) family proteins, Piezo1, and Piezo2). Moreover, ATP is released in human lung fibroblasts after mechanical stretching (Murata et al., 2014). It is also suggested that Ca^2+^ is the second messenger that activates lung fibroblast functional activity (Hinz, 2012). However, an increase in the extracellular ATP concentration is considered a “danger signal” in the pathophysiology of pulmonary fibrosis (Riteau et al., 2010). Therefore, increased Ca^2+^ in lung fibroblasts responding to mechanical stress plays a role in the progression of pulmonary fibrosis.

Fibroblasts release more pro-inflammatory cytokines and chemokines after mechanical stretching, which actively participate in regulating the inflammatory response after mechanical injury (Hawwa et al., 2011a) and promote wound healing and tissue repair. Lung fibroblasts play a key role in pathophysiological events associated with pulmonary fibrosis (Barkauskas and Noble, 2014; Riteau et al., 2010; Yamauchi et al., 2020).

#### Cancer-Associated Fibroblasts Exposed to Stretching Stimulation

Cancer is known as “wounds that never heal”, and fibroblasts activated in the tumor ECM can promote tumor inflammation and fibrosis (Kalluri, 2016); thus, they are also known as CAFs. Compared with resting fibroblasts, CAFs present a larger morphology, nuclear depression, and more branching cytoplasm (De Wever et al., 2008), and activated CAFs have stronger proliferation and migration ability (Chen and Song, 2019).

CAFs are cells with mechanical ability and are the most abundant stromal cell type in the tumor microenvironment (Bhowmick et al., 2004). They play a crucial role in cancer development and metastasis (Pereira et al., 2019). Once CAFs settle down in ECM of the tumor, they can benefit cancer cells in many aspects: 1) promote the growth and proliferation of tumor cells (Zhang et al., 2020); 2) assist in tumor-related angiogenesis (Fan et al., 2020; Lugano et al., 2020); 3) accelerate the invasion and metastasis of tumor cells (Birkbak and McGranahan, 2020); 4) cause drug resistance of some anti-tumor drugs (Wei et al., 2020). CAFs secrete soluble factors such as growth factors, cytokines, and ECM molecules during cancer progression to promote the self-differentiation of normal fibroblasts. TGF-β released by CAFs triggers the anisotropic matrix to stimulate myofibroblast differentiation of normal fibroblasts (Franco-Barraza et al., 2017). Meanwhile, excessive production of fibrillary ECM proteins and ECM remodeling by CAFs leads to cancer fibrosis, namely promoting connective tissue hyperplasia. As the stiffness of the ECM derived from CAFs increases, it usually leads to greater force (traction) exerted by cells on the matrix, as well as increased stress fibers and greater focal adhesion (Ladoux et al., 2016), thus promoting the differentiation of CAFs and supporting the progression of cancer.

There is an inverse relationship between traction stress and metastatic capacity of breast cancer cells, and the generation of force decreases with the increase of metastatic potential and capacity (Indra et al., 2011). Studies have shown that cell-matrix adhesion dynamics and traction conversion regulate tumor cell migration (Fokkelman et al., 2016). Recent studies have shown that CAFs exert physical forces on cancer cells by stimulating phenotypically pro- or anti-tumorigenic interactions that allow them to invade en masse (Yamauchi et al., 2020).

Glentis et al. (2017) found that CAFs actively reshape the basement membrane by pulling, stretching, and softening it, leading to the formation of spaces where cancer cells can migrate. By applying contractile forces, CAFs alter the organizational and physical properties of the basement membrane, allowing cancer cells to invade. CAFs promote fracture of the bone matrix in a matrix metalloproteinase-independent manner. Therefore, it has been proposed that in addition to proteolysis, the mechanical forces exerted by CAFs represent another mechanism of basement membrane rupture.

**FIGURE 2:**
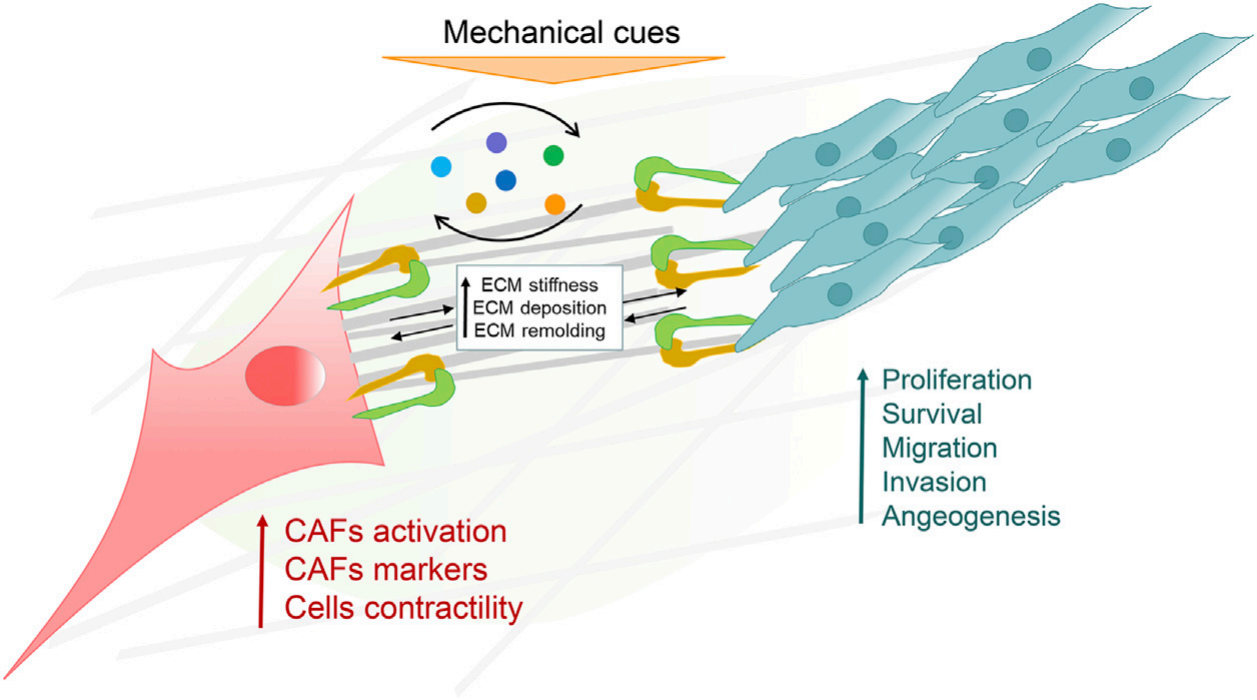
The crosstalk of cancer associated fibroblasts (CAFs) and the cancer microenvironment (CME). CAFs and cancer cells are responsible for sensing and transducing various extracellular matrix (ECM) proteins and mechanical signals in the CME. CAFs and cancer cells also effect each other’s physiological processes by releasing and receiving various factors in a paracrine manner.

In the tumor microenvironment, CAFs and cancer cells communicate through the ECM and other soluble factors to influence each other’s cellular behavior (Figure 2). Numerous mechanical signals are detected and transmitted to cells by mechanoreceptors. These receptors often communicate with ECM, where external signals are converted into physiological replies. Integrin was a well-defined mechanoreceptor that directly connected the microfilaments to the ECM and conducted signals. The 18 α and 8 β subunits heterodimerize to produce more than 24 different receptors. These heterodimers are the link between cells and the tumor microenvironment. CAFs heterogeneity drives balancing processes in tumor growth and invasion (DiPersio and Van De Water, 2019; Jang and Beningo, 2019). Tumor growing accompanied with a heterogeneous population of CAFs. The subpopulations of CAFs can promote deposition and ECM remodeling, which interact directly with tumor cells in principal and subordinate structures to help tumor cell migration and invasion. Also, it provokes angiogenic factors by mediating new blood vessel growth and yield immune cell suppression *via* ECM modification and chemokine/cytokine secretion (Yamauchi et al., 2020). Although this communication is bi-directional, as shown in the figure, we mainly consider communication as unidirectional, from CAFs to cancer cells. Mechanical forces generated by ECM remodeling induced by CAFs contribute to the invasion efficiency of metastatic cells (Menon et al., 2011).

## Concluding Remarks and Outstanding Questions

Mechanical stimulation is closely related to lung, lung cancer, and lung cancer-related fibroblasts. Mechanical strain is an important regulator of normal and abnormal lung growth and development. Mechanical forces are involved in biological processes ubiquitously (Kumar and Weaver, 2009), although the underlying mechanisms of cancer cell migration or metastasis are still not fully understood. Mechanical stimulation plays an important role in regulating fibroblasts and the ECM components within the tumor microenvironment. First, there is no literature available on the mechanisms induced in lung CAFs by mechanical stimulation. Secondly, a limited number of studies have described mechanisms induced by mechanical stimulation of other types of CAFs. However, these studies suggested that CAFs play a crucial role in tumor progression. Thirdly, the response of lung CAFs to mechanical stimulation may play a crucial role in the progression of lung cancer; thus, it will be of great clinical significance to study the response of lung CAFs to mechanical stimulation.

At present, lung biomechanics are mainly focused on mechanical ventilation, ventilation injury and fluid dynamics of vascular endothelial cells. There is a lack of research on mechanical factors affecting other lung cells (especially fibroblasts and immune cells) in normal respiration. There are few studies on the mechanism of lung tumors causing changes in lung physical properties and then affecting lung cells.

Due to the complexity of the lung cancer classification, there may be different types of lung CAFs, but once this role of mechanotransduction on lung cancer cells is recognized, this will be accompanied by further in-depth research. In the future, it will be extremely challenging and of practical significance to isolate different types of lung CAFs associated with different types of lung cancer, and elucidate the specific mechanisms involved in mechanical stimulation on tumor progression.

## Abbreviations

CAFs, Cancer-associated fibroblasts; ECM, extracellular matrix; FEV1/FVC, forced expiratory volume to full forced lung capacity in one second; TLC%, total lung capacity percentage; FEV1, forced expiratory volume in one second; PF, pulmonary fibrosis; α-SMA, osteopontin, alpha smooth muscle actin; TGF-β, transforming growth factor-β.
